# Novel 3-D action video game mechanics reveal differentiable cognitive constructs in young players, but not in old

**DOI:** 10.1038/s41598-022-15679-5

**Published:** 2022-07-21

**Authors:** Tomihiro Ono, Takeshi Sakurai, Shinichi Kasuno, Toshiya Murai

**Affiliations:** 1 Department of Psychiatry, Kyoto University Hospital, Yoshida konoe cho, Sakyo-ku, Kyoto, Kyoto 606-8501 Japan; 2 BonBon Inc., Kyoto, Japan; 3 Department of Drug Discovery Medicine, Graduate School of Medicine, Kyoto University, Kyoto, Japan; 4 Department of Psychiatry, Graduate School of Medicine, Kyoto University, Kyoto, Japan

**Keywords:** Human behaviour, Psychology, Medical research

## Abstract

Video game research predominantly uses a “one game-one function” approach—researchers deploy a constellation of task-like minigames to span multiple domains or consider a complex video game to essentially represent one cognitive construct. To profile cognitive functioning in a more ecologically valid setting, we developed a novel 3-D action shooter video game explicitly designed to engage multiple cognitive domains. We compared gameplay data with results from a web-based cognitive battery (WebCNP) for 158 participants (aged 18–74). There were significant negative main effects on game performance from age and gender, even when controlling for prior video game exposure. Among younger players, game mechanics displayed significant and unique correlations to cognitive constructs such as aim accuracy with attention and stealth with abstract thinking within the same session. Among older players the relation between game components and cognitive domains was unclear. Findings suggest that while game mechanics within a single game can be deconstructed to correspond to existing cognitive metrics, how game mechanics are understood and utilized likely differs between the young and old. We argue that while complex games can be utilized to measure distinct cognitive functions, the translation scheme of gameplay to cognitive function should not be one-size-fits-all across all demographics.

## Introduction

### Background

Psychiatry has historically relied on subjective observation for diagnosis, evaluation, and research. With technological advancement, the field has incorporated more biological measurements, including brain imaging and electroencephalography, as a part of a broader effort by researchers and psychiatrists to move towards data driven and evidence-based psychiatry^1^. Recent advancements have opened the possibility of using digital devices to collect measurements of biological responses that may in turn serve as digital biomarkers^2–5^.

In addition, the ongoing Covid-19 pandemic has made urgent the longstanding need for new methods of remotely monitoring cognitive performance and mental states due to extended periods of social isolation and uncertainty^6–11^. Remote monitoring via electronic devices can close the gap between the difficulty and costs of in-person evaluation and the restrictive realities of responding to the ongoing pandemic^6,12^.

Video games have garnered attention as a possible vehicle for digital mental health intervention due to the potential that video games’ entertainment qualities have for encouraging voluntary compliance and engagement compared to traditional interventions digital and otherwise. Because video games’ entertainment value is partly dependent on providing a challenge difficulty that matches the skill level of the player, video games also hold promise as effective inducers of cognitive training and growth^13–15^. A noteworthy first was achieved when Akili Interactive’s Endeavor obtained FDA (U.S. Food and Drug Administration) clearance for treatment of attention-deficit hyperactivity disorder through gameplay that challenges players’ impulsivity^16–18^, and long-term memory gains have been observed in adults in their sixties and seventies after playing a virtual-reality navigation game^19^. Couple the engagement and difficulty matching with the capability to accurately and precisely record behavioral data for analysis, and it is no surprise that extensive research has gone into utilizing the video game experience for cognitive measurement and training, with mixed results on whether improvement carries over to tasks different in form from the one practiced (known as “far transfer”)^19,20^. Off-the-shelf action video games have received particular attention for their potential to engage and affect a multitude of cognitive functions simultaneously. A genre having certain cognitive demands does not always translate to improvements in those domains^21^, especially when the game is not very similar to the benchmark cognitive task or when intensity is lacking^22,23^, but under certain game design constraints video games have improved even high-level cognitive functions like neuroplasticity^24^. Some studies have linked specific in-game maneuvers or conditions to observed improvement in cognitive function after extensive training^24,25^, but such reports have been relatively rare. Commercial video games satisfy the “engagement” quality that people seek in serious games, but it’s hard to interpret what exactly is happening cognitively in a video game: since they are not originally made for cognitive research, researchers cannot select the target cognitive functions at will, and interpretation is complicated by the fact that certain games resonate more with certain demographics than others in ways that are not well-documented. Presently, the profile of cognitive abilities affected by action video games is built upon decades of difficult and often conflicting reports of empirical trials, often logistically unable to quantify cognitive engagement in a video game beyond hours played or stages cleared.

### Video games as cognitive measurement tools

Researchers have also attempted to employ custom video games for measurement as well as training. Similar to how cognitive tests measure cognitive abilities insofar as performance on tests is dependent on successful use of the target abilities^26^, games can measure cognitive abilities since performance in a video game depends on successful use of the cognitive abilities most relevant to the game’s design^27–34^. Prior research has suggested that a constellation of video games can be employed to obtain measures that approximate general problem-solving skills (*e.g.* fluid intelligence *g*)^32,33^, while other applications have demonstrated that gameplay can be designed around a single cognitive function to extract biomarker-like information about a player’s cognitive abilities^35,36^, in line with age-based^35,37^ and gender-based^38^ expectations of differences in cognition. In doing so, researchers need to account for prior gaming expertise since seasoned gamers can outperform novice gamers even if the game in question is novel for all participants^38–40^.

### Design constraints and ecological validity

When devising built-for-purpose serious games, in both measurement and training, researchers rely on designing so that in-game success hinges on successful use of a focal cognitive function. In order to broaden the scope beyond single cognitive functions, some studies have employed a suite of mini-games and tasks, where individual games and tasks in the set are each designed to target a certain cognitive ability (e.g*.* memorization, fast reaction)^18,41^. However, such design constraints for interpretability come at a creative cost; simple and isolated game mechanics make for interpretable but often shallow gaming experiences, in which it’s difficult to maintain engagement for hours on end. Not only is such a creative constraint unappealing for the players, but it also makes the serious game endeavor much less appealing for game creators, hampering the development of the field.

Additionally, restricting game design around a single cognitive domain limits the ability of the game to probe cognitive domains as relevant in ecologically valid situations. Traditional cognitive tests can overestimate^42–46^ or underestimate^47–49^ cognitive decline due to them not being valid indicators of cognitive abilities as relevant to day-to-day functioning and being subject to significant practice effects. Common features of tasks made to be more ecologically valid include the use of concrete over abstract entities^43^ and allowing for a relatively complex task structure that allows for the use of compensative strategies to offset reductions in processing speed^42,46^. By constructing complex tasks in video games that involve concrete entities and multiple cognitive domains, we can profile cognitive functions in a more ecologically valid setting.

A potential boon for the serious-game field, then, is to demonstrate that interpretability and complex gameplay need not be mutually exclusive. Showing that a purpose-built serious game can be deconstructed into distinct cognitive activities while retaining complex and non-test-like gameplay will provide one roadmap for researchers and game designers alike who are burdened by sacrificing interpretability or engagement.

### Cognitive activity in a 3-D action game

Broadly, we considered two possible categories of cognitive functions that could reasonably be expected measured from an individual’s gameplay in a 3-D action game: (1) cognitive functions required for action video games in general, (2) cognitive functions specific to performing tasks in this specific game.

In their meta-analysis of action video game training, Bediou et al*.* include the following as characteristic features of action video games: (a) a fast pace of events, (b) high load on perception, motor skills, working memory, and planning skills, (c) the need to shift attention back and forth between focused attention and distributed attention, and (d) a high amount of visual distraction^21^. As such, an “overall performance” metric (such as a high score) in any action video game would be expected to partly depend on cognitive metrics that correspond to the above features, such as visual and auditory reaction, motor skills, working memory, planning, and attention control.

In addition to the cognitive functions required for playing any generic action video game, there may also be cognitive demands placed by features not universal to all video games. Among action games, there is a subgenre called stealth games where the game’s incentives are structured to reward avoiding direct confrontation with enemies, and to penalize failure to circumvent enemy detection, much like in the real-life game “hide and seek”. To our knowledge there are no studies examining stealth-related behaviors as expressed in video games and baseline cognitive abilities, but studies of hide and seek and other deception-based games point to higher-order cognitive functions such as abstract thinking and theory of mind as being crucial to performance^50,51^.

### Effects of age, gender, and prior video game experience

For each cognitive ability mentioned above, prior reports suggest the existence of demographic differences in performance, some due to biological reasons and others due to sociological reasons.

A performance advantage has been reported for younger participants over older participants and for male participants over female participants in reaction time^52,53^, motor skill^54,55^, visuospatial working memory^56,57^, planning^58–60^, and attentional control^61–63^. As such we expect overall action video game performance—which combines demands from all these cognitive functions—to be higher in men than in women, and lower in older adults compared to younger adults.

One additional consideration is the effect of prior training. In classical cognitive tests, researchers seldom worry that a significant number of participants may be highly trained in similar cognitive tests before their first visit. However, because video games from the same genre share a substantial number of features (such as the features broadly shared among action video games listed above), with video games there is the possibility that research participants have differing prior exposure to and training in similar tasks, and that therefore performance differences may represent near transfer within the same genre^40^ rather than or in addition to baseline differences in cognitive ability. As men tend to play video games more frequently than women and also report stronger preference for action video games^64^, to the extent such prior training influences results, the effect is expected to compound rather than counteract the expected male advantage in action video game performance due to cognitive function differences.

Concerning stealth behavior, to our knowledge there are no studies examining gender-related or age-related differences.

### Objectives

It is our contention that a “one video game, one cognitive function” paradigm for utilizing video games in cognitive function measurement limits how engaging the gameplay experience can be. We hypothesized that a single gameplay experience could be utilized to quantify a wide range of cognitive functions, leading to a more ecologically valid cognitive evaluation setting than prior projects. For this, we developed a novel 3-D action shooter video game. More broadly, we aimed to demonstrate the potential for richer, more immersive serious game design and a possible novel tool for quantifying how different cognitive functions work in concert towards a given task.

Based on that objective, we specifically asked these research questions:

1. How can we parametrize data from an individual’s 3-D action video game play to yield both differentiable and interpretable metrics about the individual’s cognitive function?
2. Prior reports suggest the influence of age, gender, and prior familiarity with video games on video game performance. Both underlying differences in cognitive function and differing familiarity with features common to 3-D action video games predict higher performance for younger players over older, and male players over female. To what extent does gameplay reflect these demographic variables, and how might interpretation be complicated by such factors?

Here we report results from a comparative study between selected subtests from University of Pennsylvania’s Web-based computerized neurocognitive scanning battery (WebCNP) and multiple in-game metrics from a smartphone-based 3-D action video game developed by BonBon Inc., a startup company headquartered in Kyoto, Japan. Items from a survey that asked about prior video game exposure and experience were also employed.

## Results and analysis

A total of 158 participants were a part of the study, all from Western Japan (residing in or near Kyoto). Participant ages ranged from 18 to 74 years old, with the average being 39 years old (± 15 years, s.d.). Eighty-one participants identified as being female (51.3%). The highest educational attainment of participants was as follows: two had completed middle school (1.26%), 67 had completed high school (42.1%), 20 had graduated from a 2-year college (12.6%), 47 had graduated from a 4-year college (29.6%), and 23 had completed some sort of postgraduate degree (14.6%). For video game exposure, the average hours per week reported playing video games in the past 6 months was 3.67 h ± 6.32 h (s.d.), with a heavy skew towards zero hours per week. Thirty-two participants had never played action video games before (20.2%). The age at which participants first played video games ranged from 5 to 74 years old (if the player had never played video games before, they responded with their current age) with an average age of 19.6 years old (± 18.6 years, s.d.). All participants successfully completed the cognitive battery and no issues arose from translating the instructions into Japanese. We excluded four participants due to incomplete gameplay data.

**Figure 1:**
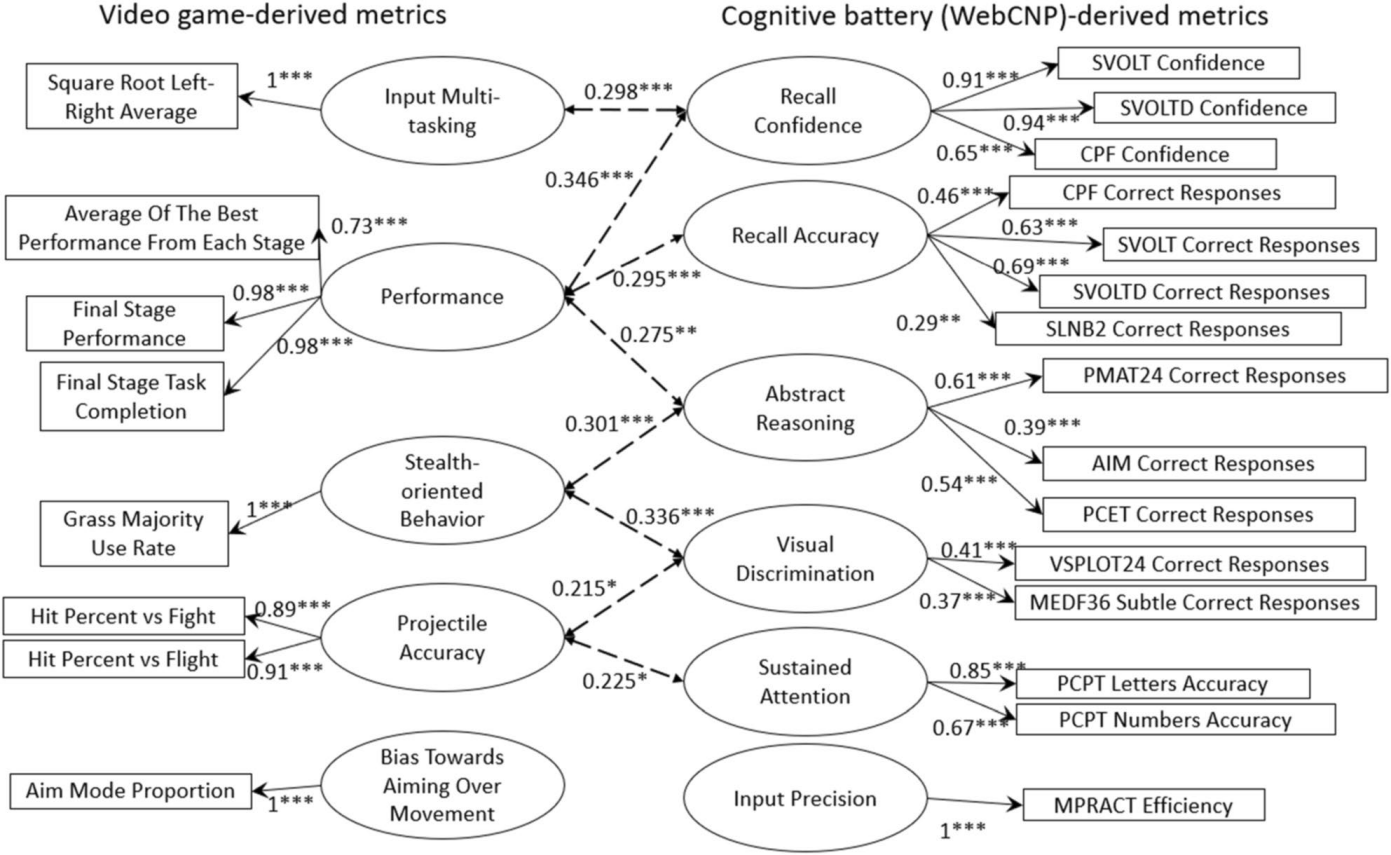
Diagram depicting the individual components used in computing latent variable metrics from video-game-based metrics (left side) and cognitive battery-based metrics (right side). All path coefficients were significant (*p < 0.05, **p < 0.01, ***p < 0.001). Standardized path coefficients shown such that the latent variable’s variance is 1.00. Dashed lines in the center between latent factors depict significant correlations (Pearson’s *r*) between video-game-derived metrics and cognitive-battery-derived metrics among those in the younger group of participants (N = 100). Older group (N = 54) correlations did not meet the significance threshold (p < 0.05). Whole-group (N = 154) inter-factor correlations not depicted; see Table 1 for values.

Results from the cognitive test were condensed through factor analysis into six parameters: Input Precision (based on The Motor Praxis Test “MPRACT” efficiency), Visual Discrimination (based on The Penn Line Orientation Test “VSPLOT24” correct responses and The Penn Emotion Discrimination Test “MEDF36” correct responses on subtle pairs), Recall Accuracy (based on the number of correct responses in The Penn Facial Memory Test “CPF”, The Short Visual Object Learning Test “SVOLT”, The Short Visual Object Learning Test Delayed “SVOLTD”, and Short Letter N-Back 2 “SLNB2”), Recall Confidence (based on how confident the participant was in CPF, SVOLT, and SVOLTD), Abstract Reasoning (based on the number of correct responses in The Penn Conditional Exclusion Test “PCET”, The Penn Matrix Reasoning Test “PMAT24”, and The Abstraction, Inhibition, and Memory Test “AIM”), and Sustained Attention (based on their performance in The Penn Continuous Performance Test “PCPT”). The right-hand side of Fig. 1 depicts the structural equation model used to aggregate the cognitive test data. We describe the contents of each test in the Supplementary Information.

**Figure 2:**
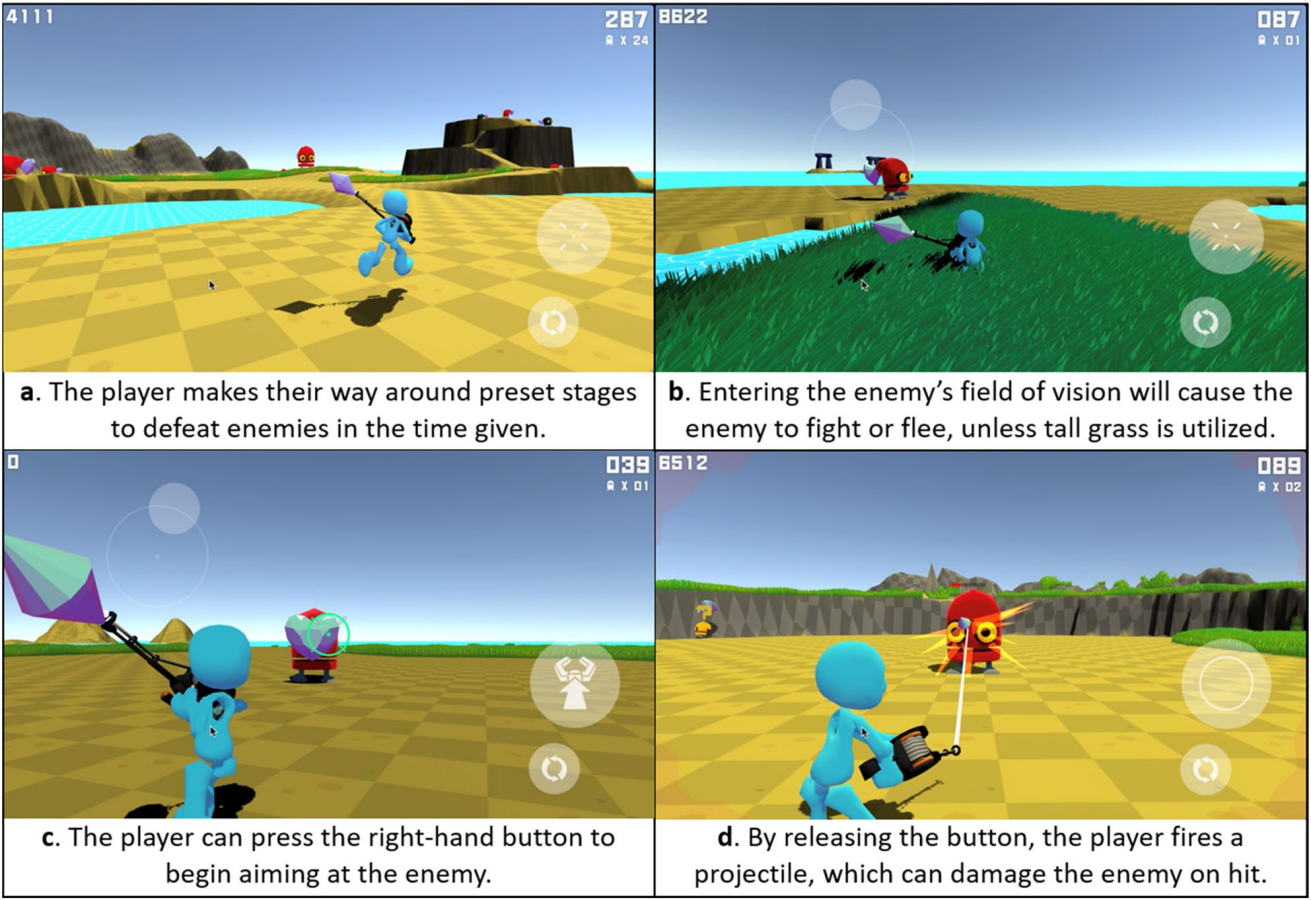
Diagram depicting common actions taken during gameplay, through screenshots of the prototype being played. How the player uses the user interface to move around and rotate the viewport to look in a specific direction (**A**) are used to calculate Input Multi-tasking. How the player utilizes stealth-assisting tall grass (**B**) is used to calculate Stealth Oriented Behavior. How long the player spends aiming (**C**) is used to calculate Bias Towards Aiming. Whether or not the player is successful in landing hits (**D**) is used to calculate Projectile Accuracy. These actions indirectly and directly aggregate into the player’s gameplay session Performance.

In-game metrics were rescaled to be z-scores and then examined for similarity, both through Euclidean distance and conceptual grouping into five parameters: Performance (from the time-based performance in the final stage, number of enemies defeated in the final stage, and the average best performance in each of the 10 main stages), Projectile Accuracy (the number of projectiles landed against the common enemy types divided by the number of projectiles shot, see Fig. 2d), Input Multi-tasking (the square root of the proportion of multi-finger maneuvers over the total maneuvers, representing how the player moved around while rotating the viewport; see Fig. 2a), Stealth-oriented Behavior (the rate at which the player utilized hiding in grass against enemies, see Fig. 2b), and Bias Towards Aiming Over Movement (from how much time the player spent aiming as opposed to simply moving, and whether or not the player moved while aiming, see Fig. 2c). The left-hand side of Fig. 1 depicts the structural model used to aggregate the in-game data. Figure 2 depicts actions often taken in gameplay.

We expected that the multiple measures obtained from various facets of gameplay would be distinct from each other and each significantly correlated to different cognitive components as measured by the cognitive exam.

However, there were significant covariances in the combined in-game metrics, the largest in magnitude being the one between Projectile Accuracy and Performance (b = 0.383, se = 0.066, p < 0.001). Since successfully completing the tasks in the game requires hitting the enemy with released projectiles, it is not surprising that Performance and Projectile Accuracy are related. Performance also covaries with Input Multi-tasking (b = 0.227, se = 0.059, p < 0.001), with Stealth-oriented Behavior (b = 0.110, se = 0.054, p < 0.05) and with Bias Towards Aiming Over Movement (b = −0.154, se = 0.055, p < 0.01). Projectile Accuracy, in turn, covaries with Input Multi-tasking (b = 0.156, se = 0.073, p < 0.05), with Stealth-oriented Behavior (b = 0.255, se = 0.075, p < 0.001), and with Bias Towards Aiming Over Movement (b = −0.151, se = 0.072, p < 0.05). Input Multi-tasking covaries with neither Stealth-oriented Behavior nor Bias Towards Aiming Over Movement, and Stealth-oriented Behavior covaries with Bias Towards Aiming Over Movement (b = −0.195, se = 0.081, p < 0.05).

As for how the in-game parameters correlate with cognitive test values, performance (conceptually, the metric that should be the closest to an aggregate reflection of cognitive abilities) is significantly correlated to all except Sustained Attention. Projectile Accuracy, which is an intermediate measure of success, is like performance but has no significant correlation to recall confidence. Input Multi-tasking (finger multi-tasking), conversely, only shows a significant correlation with Recall Confidence. The cognitive test correlations of Stealth-oriented Behavior are like those of Projectile Accuracy, but the correlation coefficient is noticeably less. Finally, the Aiming metric is not significantly correlated to any cognitive test metric. Table 1 lists the correlations, p-values, and significance levels between the five in-game metrics and the six cognitive test components, with significance denoted in asterisks.

**Table 1:** Table of correlations (Pearson’s correlation) between in-game metrics and cognitive test metrics (significance *p < 0.05, **p < 0.01, ***p < 0.001, significant cells with bold values) for the entire player sample for which metrics could be computed (N = 154 for entire sample, N = 100 for younger group, N = 54 for older group).

			In-game metrics				
			Overall performance	Projectile accuracy	Input multi-tasking	Stealth-oriented behavior	Bias towards aiming
Cognitive test metrics	Entire sample (N = 154)	Input precision	0.452*** **p = 4.04 × 10**^**–9**^	0.379*** **p = 1.23 × 10**^**–6**^	−0.035 p = 0.665	0.288*** **p = 0.000291**	−0.084 p = 0.298
		Visual discrimination	0.442*** **p = 9.22 × 10**^**–9**^	0.432*** **p = 2.27 × 10**^**–8**^	−0.015 p = 0.857	0.386*** **p = 7.87 × 10**^**–7**^	−0.088 p = 0.282
		Recall accuracy	0.486*** **p = 1.72 × 10**^**–10**^	0.427*** **p = 3.30 × 10**^**–8**^	0.106 p = 0.192	0.288*** **p = 0.000285**	−0.019 p = 0.818
		Recall confidence	0.243** **p = 0.00236**	0.149 p = 0.065	0.218** **p = 0.00670**	−0.024 p = 0.765	−0.027 p = 0.135
		Abstract reasoning	0.552*** **p = 1.19 × 10**^**–13**^	0.492*** **p = 9.69 × 10**^**–11**^	0.051 p = 0.533	0.392*** **p = 4.88 × 10**^**–7**^	−0.071 p = 0.381
		Sustained attention	0.043 p = 0.598	0.109 p = 0.180	0.028 p = 0.729	0.088 p = 0.277	−0.084 p = 0.298
	Younger subset (N = 100)	Input precision	0.105 p = 0.298	0.003 p = 0.975	−0.114 p = 0.258	−0.058 p = 0.565	−0.025 p = 0.803
		Visual discrimination	0.141 p = 0.161	0.215* **p = 0.032**	−0.004 p = 0.971	0.336*** **p = 0.000623**	−0.025 p = 0.803
		Recall accuracy	0.295** **p = 0.00289**	0.139 p = 0.167	0.132 p = 0.191	0.186 p = 0.063	0.000 p = 0.997
		Recall confidence	0.346*** **p = 0.000419**	0.095 p = 0.349	0.298** **p = 0.00**260	−0.119 p = 0.238	0.082 p = 0.415
		Abstract reasoning	0.275** **p = 0.00571**	0.189 p = 0.060	0.070 p = 0.489	0.301** **p = 0.00237**	0.026 p = 0.799
		Sustained attention	0.074 p = 0.465	0.225* **p = 0.024**	0.064 p = 0.524	0.163 p = 0.104	0.086 p = 0.397
	Older subset (N = 54)	Input precision	0.107 p = 0.443	0.037 p = 0.791	−0.097 p = 0.485	0.169 p = 0.223	0.071 p = 0.608
		Visual discrimination	0.262 p = 0.055	0.121 p = 0.382	−0.223 p = 0.104	0.062 p = 0.655	0.000 p = 0.998
		Recall accuracy	0.180 p = 0.194	0.269 p = 0.050	−0.057 p = 0.680	0.050 p = 0.719	0.194 p = 0.160
		Recall confidence	0.097 p = 0.486	0.232 p = 0.091	−0.002 p = 0.988	0.105 p = 0.448	0.225 p = 0.103
		Abstract reasoning	0.217 p = 0.115	0.185 p = 0.180	−0.161 p = 0.245	0.096 p = 0.492	0.067 p = 0.632
		Sustained attention	0.178 p = 0.198	0.041 p = 0.769	−0.134 p = 0.335	−0.125 p = 0.369	0.090 p = 0.517

Although Projectile Accuracy and Stealth-oriented Behavior reflect similar cognitive abilities, all other metrics have different correlations to cognitive test metrics and are distinct from each other as previously noted, demonstrating that it is possible to extract distinct metrics from various facets of gameplay that each contain different information about the player’s cognitive processes.

Next, we examined whether gameplay performance significantly differs based on sociodemographic data, specifically across age and gender. We found that there is a pronounced gender disparity in Performance (with male players scoring higher than female players) both in the average Performance level and within each age group, and that the trend seen in Performance with increasing age group differs by gender. Specifically, the performance of female players is comparable between those in their teens, twenties, and thirties, with a sharp drop-off in performance in their forties, while for men, the sharp drop-off in performance happens between players in their forties and fifties, approximately a decade later than the trend with female players. A similar trend was found in participant’s self-rated affinity for action games and recent video game exposure. For men, we categorized all those under the age of 50 into the younger group, whereas for women we categorized all those under the age of 40 into the younger group, resulting in N = 100 participants in the younger group and N = 54 participants in the older group. Figure 4 displays the model-fitted performance for each gender and age group, computed from the final model (Model 8) as seen in Table 2. Post-hoc generalized linear model tests indicated significant difference between genders (t = 4.241, p < 0.001) and age group (t = 2.242, p = 0.026). The difference between young men and old women was significant (t = 5.595, p < 0.001), but not between old men and younger women (p = 0.951).

**Table 2:** Table of multiple linear regression models with in-game performance as the dependent variable (significance shown on regression coefficients with standard error in parentheses below; *p < 0.05, **p < 0.01, ***p < 0.001).

Model	Dependent variable: performance							
	(1)	(2)	(3)	(4)	(5)	(6)	(7)	(8)
Current Age	−0.049***	−0.049***		−0.043***	−0.046***	−0.047***	−0.044***	−0.034***
	(0.004)	(0.003)		(0.005)	(0.005)	(0.003)	(0.006)	(0.006)
Gender (0 = Not female, 1 = female)		−0.707***			−0.532***	−0.613***	−0.567***	−0.462***
		(0.098)			(0.118)	(0.092)	(0.109)	(0.109)
Action video game affinity (7-point Likert scale)			0.222**	0.160*	0.073			
			(0.078)	(0.061)	(0.060)			
Input Multitasking (z)			0.400***	0.274***	0.237***	0.235***	0.238***	-0.057
			(0.074)	(0.059)	(0.055)	(0.046)	(0.047)	(0.093)
Recent Video Game Exposure (z)			0.150*	0.082	0.041			
			(0.071)	(0.056)	(0.052)			
Generation (0 = older; 1 = younger)							0.149	0.445*
							(0.188)	(0.199)
Input multitasking * generation								0.408***
								(0.112)
Intercept	1.913***	2.253***	0.171*	1.758***	2.099***	2.156***	1.883***	1.251**
	(0.161)	(0.147)	(0.075)	(0.189)	(0.191)	(0.138)	(0.371)	(0.397)
Adjusted R^2^	0.509	0.633	0.295	0.575	0.637	0.685	0.684	0.708
F statistic	159.834*** (df = 1; 152)	133.116*** (df = 2; 151)	17.578*** (df = 3; 116)	41.271*** (df = 4; 115)	42.692*** (df = 5; 114)	111.744*** (df = 3; 150)	83.758*** (df = 4; 149)	75.147*** (df = 5; 148)

Model 1 suggests that performance and age are associated, while Model 2 suggests that there is a gender gap in performance separate from age-related variance. Models 3–6 examine which measures—both in-game and external—provide information about prior video game exposure while controlling for age and gender. Models 7–8 suggest that the relationship between multitasking input and performance is vastly different between younger and older players.

*p < 0.05; **p < 0.01; ***p < 0.001.

**Figure 3:**
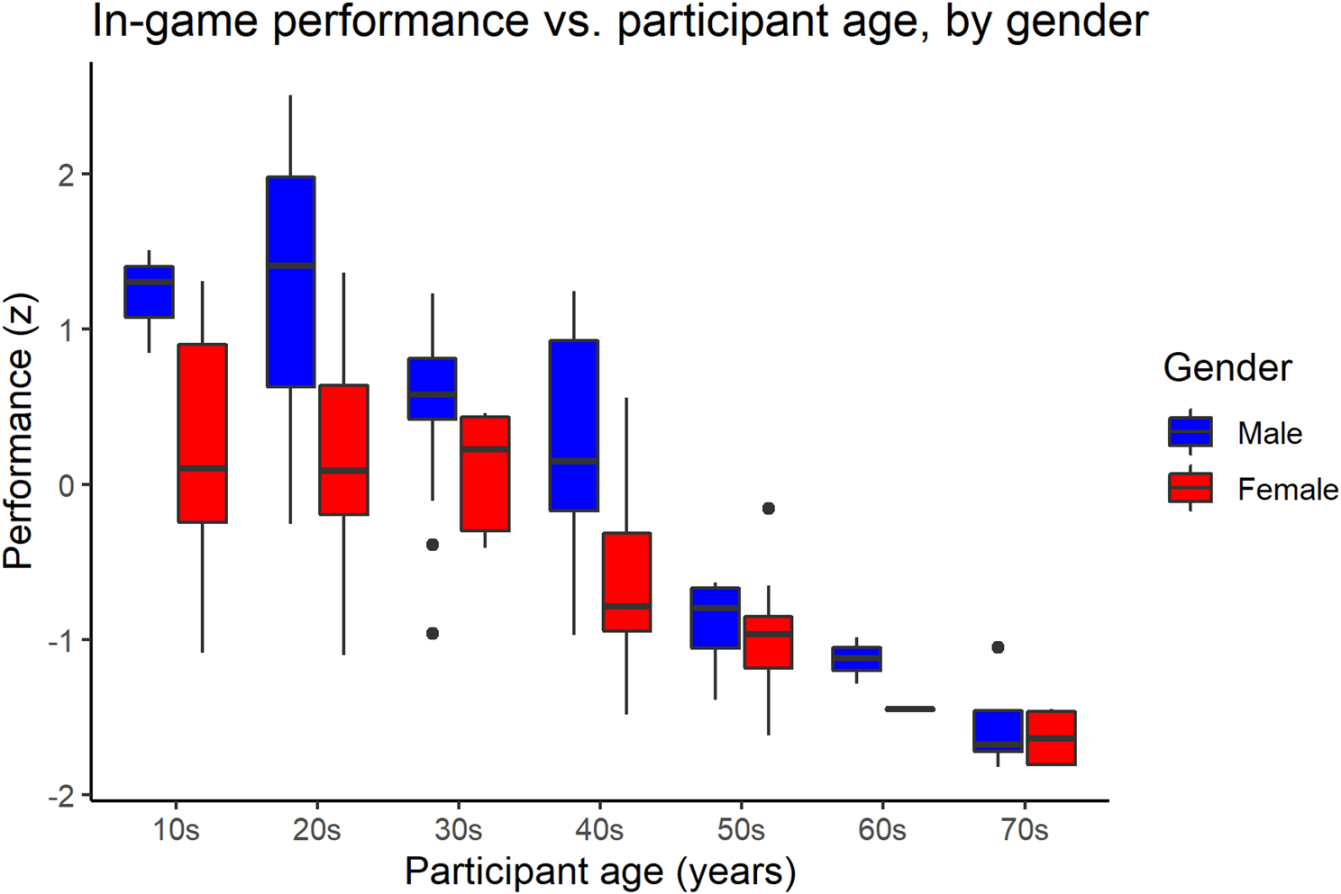
Box plot of player performance grouped by age group and separated by gender. Bold lines indicate median value; box bounds represent first and third quartile, while dots represent outliers based on interquartile range. In both genders average performance is comparable across younger age groups but is noticeably lower beginning with a specific age group (40 s for women, and 50 s for men).

Figure 3 depicts the distribution of performance scores for players in each age group.

**Figure 4:**
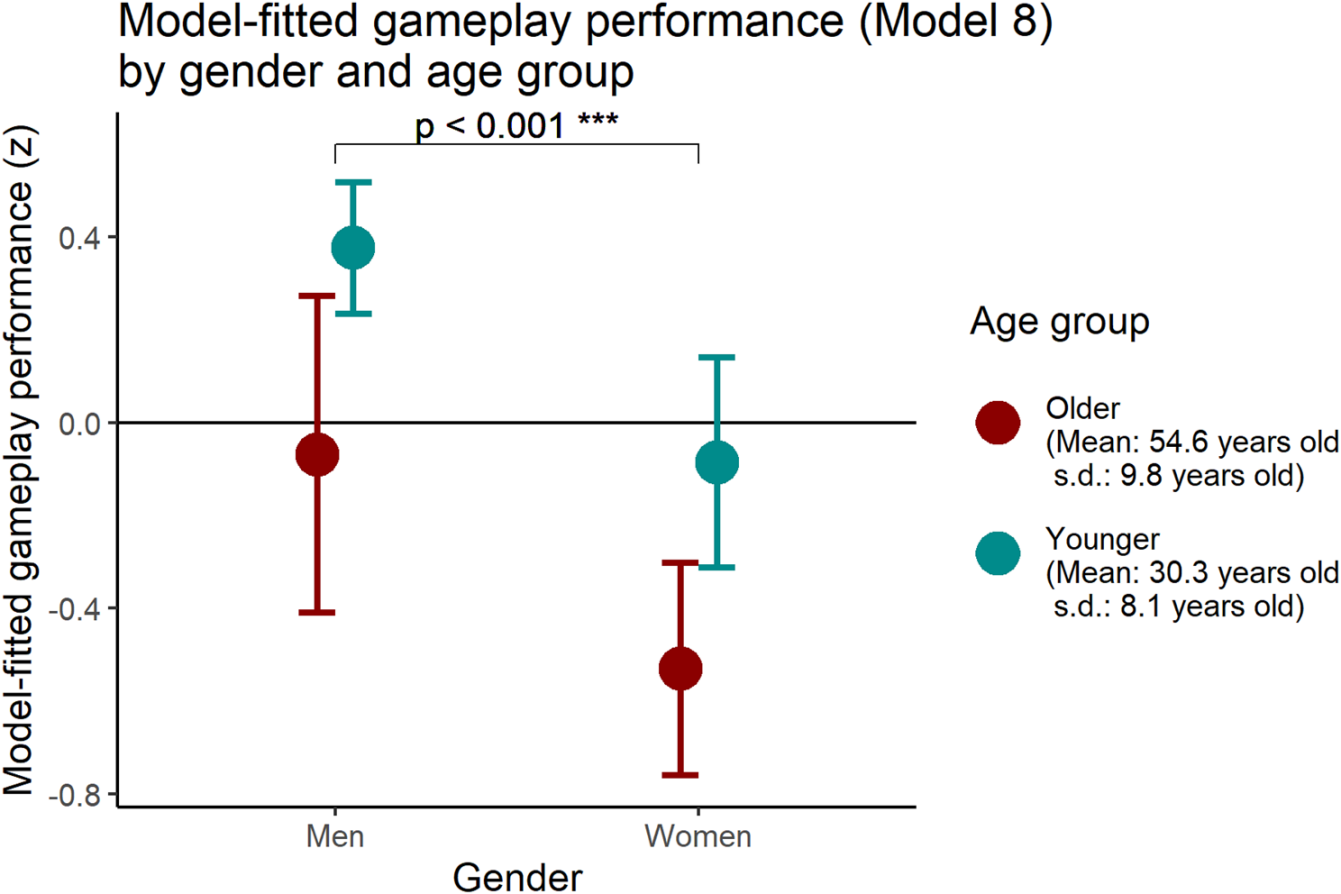
Model-fitted in-game performance as a function of age group and gender, controlling for input multi-tasking and actual age (age was set to 40, the mean, for the comparison). Final model (model 8) used.

Regressing performance to age (Model 1), then to both age and gender (Model 2) confirms that being younger and being male, both had substantial main effects on performance (adjusted R^2^ = 0.633 for Model 2 with age and gender). Table 2 shows the regression coefficients for each model (Fig. 4).

Having demonstrated that there is a clear difference in performance between groups separated by age and gender, we examined whether the difference in performance was due to differing levels of familiarity with the video game medium or due to an underlying decline in cognitive ability. To examine this, we utilized the participants’ first age of exposure to video games and hours per week playing video games in the past 6 months, and their affinity towards action video games, all items from the post-game questionnaire. Additionally, we examined to what extent player input tendencies predicted overall performance.

The “Input Multi-tasking” metric from gameplay behavior is a measure of how often the player played using both thumbs at once. This allows players to both move the avatar and swing the viewport around simultaneously. The game is entirely playable and completable without using this feature (especially because altering the viewport is also possible by tilting the device). However, using both fingers at once is also how players accustomed to playing similar 3-D action video games maneuver their controls. Because this behavior of utilizing both controls at once does not fundamentally advantage the player in this game’s strategy, we took this to be an empirical proxy of prior exposure to similar videogames rather than a strong indicator of one of the tested cognitive abilities.

Table 2 (Models 3–6) shows the model-building process for evaluating metrics related to game familiarity. Regressing in-game performance reveals that all three of these metrics are separately predictive of in-game performance in Model 3, from action game genre affinity (b = 0.222, se = 0.078, p < 0.01) to Input Multi-tasking (b = 0.400, se = 0.074, p < 0.001), to recent video game exposure (b = 0.149, se = 0.071, p < 0.05). However, upon introducing player age in Model 4 (Table 2), recent video game exposure ceases to be a significant predictor of performance (b = 0.082, se = 0.056), and upon further introduction of player gender as a variable in Model 5, action game preference ceases to be a significant predictor as well (b = 0.073, se = 0.060). Removing the non-significant parameters, we arrive at Model 6; only Input Multi-tasking remains as a significant predictor (b = 0.236, se = 0.047, p < 0.001) of player performance while controlling for player age and gender.

However, we see that even when controlling for Input Multi-tasking differences, age and gender remain considerable predictors of in-game performance, thereby refuting our hypothesis that age-related and gender-related differences are predominantly explained by differences in familiarity with the video game medium rather than other age-related qualities (such as cognitive decline) and gender-related qualities (such as differences in average spatial abilities unrelated to video games^33^). It appears that though familiarity with the touchscreen device and the video game paradigm (as measured by optional dual finger for input) is a vital component of overall performance, it is by no means enough to explain the clear gender differences and age-related decline seen in performance.

Recalling that performance displayed a discontinuity depending on whether the participant belonged to below or above a divide (Fig. 3), we examine whether the relationship between performance and Input Multi-tasking (while controlling for age and gender) differs between the two generational groups.

Adding the grouping variable to the performance model in Table 2 (Model 7) yields no new useful information, as the grouping alone does not predict performance any better than the combination of Input Multi-tasking, age, and gender. However, allowing Input Multi-tasking and grouping to interact in Model 8 reveals that the main effect of Input Multi-tasking becomes non-significant (b = −0.058, se = 0.093, p = 0.09), and the interaction becomes significant (b = 0.410, se = 0.112, p < 0.001). Meanwhile, age and gender continue to have significant (p < 0.001) main effects, and the grouping appears to have a significant main effect (b = 0.445, se = 0.199, p = 0.026) separate from the other parameters.

**Figure 5:**
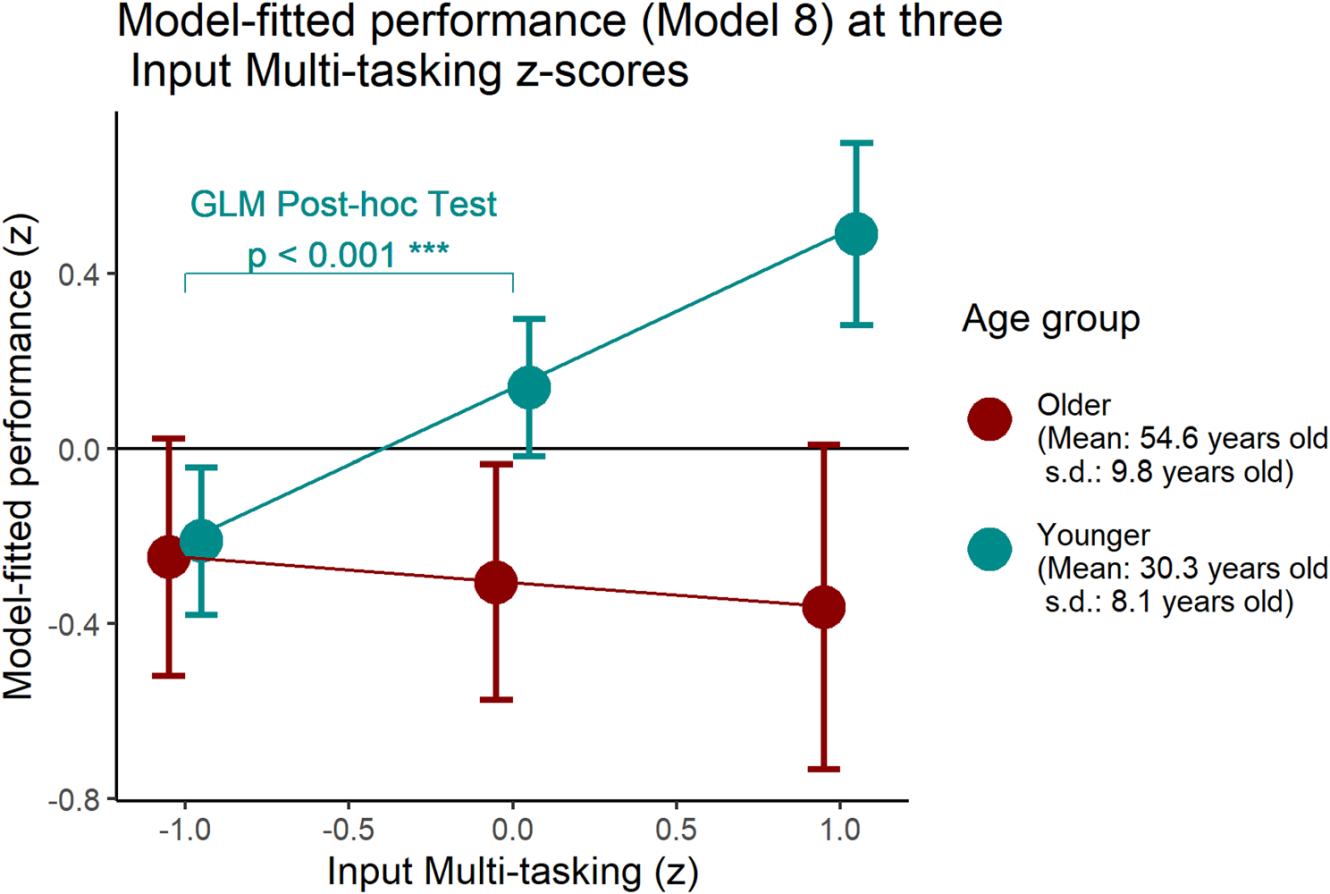
Performance as a function of Input Multi-tasking, separated by grouping (as previously computed).

Plotting model-fitted Input Multi-tasking by performance reveals that the relationship between Input Multi-tasking and performance is quite different for those below and those above the divide, as seen in Fig. 5. For the younger group, a higher z-score on Input Multi-tasking tendencies predicts higher performance, suggesting that the increased propensity to maneuver with both fingers at once reflects their accumulated expertise with similar games; for the older group, no such relation exists.

If finger multi-tasking (Input Multi-tasking) and game performance are related in quite different ways depending on the age group, what does this imply about the relationship between other measurements of the player and in-game metrics?

Going back to comparing the associations between game metrics and cognitive test metrics for the whole group and for the younger group (Table 1), we find that the relations between game-derived factors and cognitive test-derived factors differs depending on who is included in the set. While the whole-group comparisons suggested that in-game metrics were related to almost all cognitive tests, the younger group comparisons suggest that Performance is significantly correlated with just abstract reasoning (p < 0.01), recall accuracy (p < 0.001), and recall confidence (p < 0.01); that Projectile Accuracy is reflective of Sustained Attention (p = 0.024) and Visual Discrimination (p = 0.032), and that Input Multi-tasking is related to Recall Confidence (p < 0.01, as before). Stealth-oriented Behavior is related to Abstract Reasoning (p < 0.001) and Visual Discrimination (p < 0.01) and aiming is unrelated to the various cognitive parameters. With the older group, however, all links between cognitive tests and in-game performance vanish, as do a few of the within-game and within-cognitive test correlations (data not shown). Figure 1 shows the significant correlations between game-derived and cognitive-test-derived metrics for the younger subset only as dashed lines in between latent variables (in the older subset, there were no significant correlations between the game-derived and cognitive-test-derived metrics).

Amongst younger players, different metrics drawn from different facets of gameplay each correspond to a combination of cognitive tests (except for bias towards aiming over moving) and are statistically distinct (at the very least, less interrelated than the cognitive test components). This suggests that a different analysis approach is necessary for older versus younger players of video games, and possibly that the same video game cannot reliably reflect the same cognitive abilities across generational divides.

## Discussion

The results demonstrate that in-game behavior metrics correlate to distinct tests contained within the traditional cognitive battery employed here, with the cognitive ingredients of each game metric being more defined and unique among younger players. Our findings are cause for optimism regarding using different facets of a single gameplay to characterize distinct cognitive behaviors in players, but simultaneously caution against a one-size-fits-all approach, as the relation between game mechanics and cognitive tests becomes unclear in older players.

Having demonstrated that the various facets of gameplay are tied more closely to cognitive measures in the younger generation (henceforth the digital generation), further examination of the individual correlations reveals that the connections are (for the most part) sensible based on what ought to be happening in those gameplay moments. Performance being related to abstraction, recall confidence, and recall accuracy is sensible as successfully completing the in-game task requires accurately comprehending environmental cues and strategizing towards optimizing one’s movements based on what enemies are remaining and nearby. Projectile Accuracy being related to sustained attention and visual discrimination are also sensible, considering that successfully aiming the projectile at an enemy (especially from afar) requires minute adjustments in reticle positioning and possibly extended waiting until the enemy moves or orients in a certain way. Input Multi-tasking being related to recall confidence is interesting, but the conceptual relation is not immediately obvious. Stealth-oriented Behavior being related to abstraction and visual discrimination are sensible as well since the enemy’s detection radius is not visually obvious and must be extrapolated from the player’s experience with other enemies; visually discerning the orientation and distance of enemies from afar requires detecting minute differences in the displayed avatar. The aiming parameter in-game remained independent from all other metrics, in-game or otherwise, and may represent a cognitive activity or tendency that we were unable to capture in the metrics we employed in the present study.

As expected from previously reported underlying differences in cognitive function as well as differences in prior gaming exposure, on average young male players marked the highest performance while older female players recorded the lowest performance metrics (Fig. 4). This in itself is not surprising: On average, men perform better than women on 3-D navigational tasks in video games^38^, and younger players perform better than older players on fast-paced action games^35^. We used input multitasking as a measure of prior exposure to gaming—which seems reasonable, given that it predicted in-game performance much better than self-reported hours spent gaming and self-reported affinity towards action video games—and significant main effects from age and gender remained (Table 2, models 7–8). This suggests that while in-game performance reflected prior gaming experience to some extent, the difference in performance between the demographic groups also represents differences in underlying cognitive ability to an extent. Demonstrating that both are present in the overall performance metric is important because video game exposure also differs by demographic, it can be difficult to separate performance differences from prior exposure and from differences in relevant cognitive domains. Much about the relationship between prior gaming experience and performance in a new video game remains to be known: past video game experience (alongside traditional cognitive abilities) has been shown to selectively predict learning and performance in novel video game experiences within the same game genre^40^, but further research is needed to elucidate the mechanism by which prior video game experience influences learning in new video games and whether it is possible to distinguish experience-aided learning from general cognitive ability-aided learning. There is also research to suggest that age of first exposure to video games, rather than recent exposure to video games, is a more accurate predictor of certain cognitive abilities, possibly due to the influence of video games being introduced during a period of developmental neural plasticity^39^.

Furthermore, our present study demonstrated that even links between gameplay and cognitive ability prominent in those able to navigate the game (younger generation in our case) are not guaranteed in those unfamiliar with the game (older generation in our case). If video-game-based biomarkers are to become feasible, proposed applications will need to be validated with specific demographic groups. The present study’s game was designed to be sufficiently complex to prevent ceiling effects among younger players, but this resulted in the performance of older players being restricted to a lower, narrower range. Conversely, we can easily imagine a video game that is simple enough to be approachable for video-game-novice older adults that is too simple to pick up on the differences between experienced younger video game players. Just as we would not advise overlooking differences in video games even within the same genre, we would not advise taking it for granted that a video game has the same cognitive profiling ability against all demographic groups.

Complex video games are correspondingly more difficult to design and validate, requiring close collaboration between scientists and game engineers; and unless they are built to-purpose, commercial video games will often be dilute in their cognitive targeting^15^ and/or not allow for the type of granular data collection needed to examine game mechanics separately. Despite those difficulties, we assert that complex video games have their place in evaluating cognitive behaviors and abilities, especially when characterizing cognitive abilities not in isolation but in concert. Real-life tasks do not necessarily have rigid structures that employ a single cognitive ability. Complex video games have the potential to capture cognitive processes and decision making amidst uncertainty much more realistically than tasks with artificially reduced complexity can.

### Limitations

As has been noted by others^15^, this first-pass exploration of video game play components and whether they can be used to model cognitive constructs ought not be considered complete in terms of validating this serious game. The present study had several limitations, of which we list some notable ones below.

In terms of study design, the present report was limited in at least three aspects. First, because we did not shuffle the activity sequence, we cannot rule out the possibility that the order of activities influenced the results. More specifically, there is the possibility that mental exhaustion (and variance in it) affected performance in the subsequent video game; a future area of focus could be to examine the degree to which mental depletion affects video game play. Second, we drew our entire sample from people in Western Japan, so the racial-ethnic and linguistic diversity of the participants was near nonexistent. It remains to be seen whether these associations between gameplay and cognitive tests hold across different cultural demographics than that of our sample. Third, we did not perform psychiatric screening on our participants, so there is the possibility that an unreported, unobserved psychiatric condition lurked in the results, particularly in the older group.

In terms of game design, there were also three limitations as well. First, because the tutorial stage was relatively short and did not explain every intricacy of the game, we cannot rule out the possibility that certain mechanisms in the game remained undiscovered by certain players, affecting their overall performance. For example, there are enemy types that appear in the main stages but not in the tutorial. The general principle of how to deal with enemies is explained, but the peculiarities of each enemy are not, and so a player not versed in video games may not figure out how to deal with a particularly tough enemy within the short 10-stage cycle of the experiment. Second, the game did involve the concept of limited health for the player but there was no penalty for being damaged beyond that capacity apart from being sent back to the initial position on the outskirts of the stage. Since it was possible to repeatedly fail and try again, certain players may have not perceived the threat that enemies pose as well as we would have liked. Third, because we used an action video game, there was likely an advantage to people who have played similar games before. Prior action video game experience is predominantly found in younger generations, so older adults were effectively placed on a steeper learning curve by default. Studies have suggested that there are significant cognitive gains to be reaped in populations thought to experience difficulties in a certain domain, such as visually impaired youth, dyslexic patients, individuals with Down Syndrome, and older adults^19,65–67^, so the fact that video-game-novice older adults yielded more unpredictable data is unsurprising: we cannot say for sure where they were on their learning trajectory when the game ended, and for some players the learning may not have even begun.

Lastly, in terms of analysis, we recognize two key limitations.

In grouping cognitive tests or in-game measurements into latent variables, we prioritized interpretability based on the cognitive functions that the component metrics were thought to represent. This resulted in some of the constructs in the structural model not having the strongest relations to their underlying metrics, such as Visual Discrimination on the cognitive test side having only a 0.41 and 0.37 factor loading with the line orientation test and the emotion discrimination test, respectively. Further studies and analyses may be warranted to determine if relating these tests in isolation to game elements would yield stronger interpretations.

Additionally, the present study only involved quantitative comparisons and focused on overall averages or measurements at certain points in game play. This limited our ability to examine how players of different expertise levels and demographics may employ different strategies or exhibit different behaviors and emotional states during game play. Similarly, by focusing on accuracy scores rather than efficiency or reaction time in the cognitive tests, we leave out characterizations of participant strategy when performing cognitive tasks. Cognitive strategies and emotional states might both affect the results highlighted in this study and be informative outcomes themselves; this makes them worthy targets of further research.

## Conclusion

Video games hold promise for bringing the best of entertainment and psychometrics together; past studies have explored characterizing single cognitive abilities using a game or several games. Here, we explored the feasibility of characterizing multiple cognitive components through metrics from different facets of a single gameplay experience. The metrics collected from different processes within the game were shown to be distinct and each uniquely related to metrics aggregated from a traditional cognitive battery administration. We found that age and gender both have significant effects on overall performance, with a striking difference between those born after the advent of video games and those born approximately before. Furthermore, we found that the connection between in-game metrics and cognitive measures was more organized and defined in the younger group but unreliable in the older group. Thus, our game was useful in characterizing a constellation of cognitive abilities in younger players, but we failed to define what the same parameters meant in older players.

Given this tension between video game design and which cognitive abilities it will activate in whom, why did we opt to design a complex video game with multiple moving parts instead of a more strictly defined and analytically simple gamified cognitive test? We see the cognitive research potential of video games not just in strictly defined tasks but also in complex richness of professionally designed gameplay. We thus believed it worthwhile to explore the feasibility of characterizing the cognitive processes involved in complex, multi-faceted gameplay. Our hope is that the present study informs and motivates further collaboration between researchers and professional video game designers in utilizing complex games in cognition research.

## Methods

### Participants

Eligible participants were over 18 years of age, and capable of playing the 30-min video game session. We excluded those who did not meet the age threshold, reported being sensitive to flashing lights, reported being prone to motion sickness, or were otherwise unable to complete the tasks in the study. The participants resided in Kyoto, Japan or in its vicinity.

### Materials

We obtained game-based metrics from a novel 3-D action video game software developed by BonBon Inc. of Kyoto, Japan. We describe the game design in detail in the Supplementary Information. We defined the in-game measurements to be collected and compared based on extensive discussions with the game designers and developers, to balance interpretability and feasibility, and then further narrowed metrics down based on whether they yielded distributions tolerable for regression in an internal study prior to the present experiment.

In order to profile participants’ cognitive abilities for comparison against game metrics, we used WebCNP, a web-based computerized neurocognitive scanning^26^. We excluded tests centered around verbal memory, as the test was with English words and English was not the primary language for our participants. Additionally, we examined the tests in terms of accuracy rather than efficiency or response time, as the first test of motor praxis score was functionally a reciprocal response time, and with one exception the response time measurements for the other tests could all be explained by one latent variable, which in turn was essentially the “Input Precision” factor we note in our structural equation model. In the Supplementary Information we provide a brief description of the subtests used, as well as a description of the response time factor in relation to the Input Precision variable.

### Institutional review

The study protocol received approval from The Kyoto University Psychological Science Unit Ethics Committee (Identifier 2-P-30) and was performed in accordance with relevant guidelines and regulations.

### Procedure

We called for participants via social media and by word-of-mouth in the local community, with specific emphasis on video game experience not being a prerequisite. The initial call included a description of the study protocol, aims, and conditions. Once on-site, the participant received a verbal explanation of the terms and gave their written informed consent. The cognitive exam portion took about 60 min for twelve subtests. Trained staff verbally translated the on-screen instructions into Japanese according to a predetermined script and answered any questions concerning the subtests before beginning. After the cognitive exam portion, participants received a 5-min break, and then played the video game for one tutorial stage and ten data-collecting stages, which took 20–30 min. Staff answered questions about gameplay only during the tutorial stage. After gameplay, the participant responded to a short questionnaire about their demographics and prior video gaming experience. After confirming their participation consent, participants received a gift card compensating them for their time and ended their participation. All participants provided written informed consent at the beginning and confirmed their consent at the end.

### Analysis

We checked in-game metrics for ceiling and floor effects, and for any bimodality. We combined both in-game metrics based on common conceptual features and results for exploratory factor analysis, and then checked their covariance to confirm that they were differentiable factors. Metrics from the Penn Computerized Neurocognitive Battery were summarized into factors through confirmatory factor analysis^68,69^. Finally, we explored the relationships between in-game metrics and sociodemographic data as well as prior video game experience.

We performed all statistical analysis using the R statistical programming language^70^. Statistical significance tests were conducted with an alpha level of 0.05 and all tests where applicable were two-tailed. Plots visualizing the data were created with the *ggplot2* package^71^.

## Supplementary Information

Supplementary Information.

## Data Availability

The datasets generated during the current study are not fully publicly available due to conditions on the handling of raw data, but anonymized datasets are available from the corresponding author on reasonable request.
