# Supplementary material for: Novel 3-D action video game mechanics reveal differentiable cognitive constructs in young players, but not in old
Source: Sci Rep. 2022 Jul 21;12:11751. doi: 10.1038/s41598-022-15679-5 (PMC9304325; doi:10.1038/s41598-022-15679-5)
Supplement: Supplementary file 1 — Supplementary Information. [file 41598_2022_15679_MOESM1_ESM.pdf]

Novel 3-D action video game mechanics reveal differentiable cognitive constructs in young players, but not in old

Tomihiro Ono[1,2*], Takeshi Sakurai[2,3], Shinichi Kasuno[2], and Toshiya Murai[4]

[1]Department of Psychiatry, Kyoto University Hospital, Kyoto, Japan.

[2]BonBon Inc., Kyoto, Japan.

[3]Department of Drug Discovery Medicine, Graduate School of Medicine, Kyoto University, Kyoto, Japan

[4]Department of Psychiatry, Graduate School of Medicine, Kyoto University, Kyoto, Japan

[*]Correspondence: tomiono@gmail.com (+81 70-8326-2205), Yoshida konoe cho, Sakyo-ku, Kyoto, Kyoto 606-8501, JAPAN.

Supplemental Note – Description of individual metrics in video game and cognitive test

Video game

The video game software used in the study was developed by BonBon Inc., of Kyoto, Japan, between April 2020 and May 2021. It is a 3-D action video game played on a handheld touchscreen smart device (a smartphone, in this study). Players interacted via two input modes: touch input—dragging, pressing, and releasing on the screen with one or both thumbs—and gyroscope input—altering the direction of view within the video game by tilting the physical device, though the game could be played without using this input. In the game sequence, the “player” avatar is transported through a series of stages. Each stage has a visible time limit and several “enemy” avatars that wander on the virtual terrain based on preset algorithms. The task given to the player is to “defeat” as many enemies possible within the allocated time. There were 85 enemies total, of which 2 were in the tutorial, and 34 were in the final stage.

In the game, there are three distinct trade-offs.

The overarching one is the time tradeoff. Players are shuttled through the stages regardless of their success level in each. If they defeat all enemies in a stage before time is up, they are transported to the next stage immediately. If the player runs out of time, the remaining enemies are marked as incomplete, and the player is warped to the next stage. The time limit was scaled depending on the initial number of enemies in the stage and ranged from 1 minute to over 5 minutes.

When aiming at an enemy, a relevant tradeoff is distance. When the player presses on an on-screen button to enter the “aim” mode, the in-game camera zooms in slightly and an on-screen reticle shows where the projectile will be launched towards when the player releases the button. Within a certain range the on-screen reticle will tell the player whether a hit is guaranteed, but to be close enough to the enemy for the predictor to be active opens the player up to risks of being spotted and/or attacked. Farther away, the player is safer, but it is more difficult to aim the projectile precisely.

When navigating through the stage, a relevant tradeoff is stealth. The enemy algorithm has a sight range (approximately a 60-degree lateral range fanning out from the front eyes of the enemy), and the enemy will change their behavior to fight or flight when the player enters their field of vision. The detection range is limited, but large enough so that the player can’t easily snipe the enemy from outside possible detection. There are plots of grass in certain stages on the terrain that allow the player to evade detection while in them. The player chooses between using extra time to navigate outside of detection range (by detouring or waiting in plots of grass) or to move towards the enemy risking detection.

When the player successfully lands a projectile on an enemy, a visible health bar will decrease by a set amount; once the health bar is emptied, the enemy is defeated. Each enemy has a “critical” area on its backside that allows the player to defeat the enemy in one hit. This allows players who take time to successfully aim and hit the back of an enemy to recuperate the preparation time, since being detected or hitting the enemy from the front would take more shots and thus more time.

Based on these trade-offs, we devised metrics that described player behavior relating to these in-game actions through a series of conversations with the video game designers and developers. For example, the stealth trade off (taking time to hide in grass plots versus taking the shortest path through open space) was operationalized as the ratio of time spent in grass versus time spent in the open while the player was within actionable range of a given enemy (roughly equivalent to the maximum reliable shot distance, or twice the detection radius of the enemy).

Cognitive Battery

For obtaining cognitive component metrics, we used WebCNP, a computerized neurocognitive battery developed at University of Pennsylvania [1]. We excluded tests centered around verbal memory, since we drew participants from a non-English-speaking

population and included those we presumed would have some correlation to in-game cognitive processes. Below, we briefly describe each of the tests used in our analysis, grouped by their common features. For each test, we included the metric relating to accuracy (as opposed to efficiency or reaction time) in our analysis model, as our research questions pertained more to the overall cognitive domains being used in a given cognitive process. A follow-up analysis of the reaction times for each test revealed that the reaction time z-scores were predominantly representative of one underlying factor, which in turn was effectively a reciprocal of the Motor Praxis Test score due to how that particular test is structured.

The one exception was reaction times in the Penn Matrix Analysis Test, which was related to accuracy on said test. Since both the Motor Praxis Test and Penn Matrix Analysis Test scores were already included in our structural equation model, we opted to not include a redundant reaction time factor, though we acknowledge that such analysis would be merited in a more in-depth analysis of the cognitive strategies that participants might be employing both in-game and in the cognitive tests (such as speed versus accuracy).

Tests of input precision

The Motor Praxis Test (MPRACT) involves a series of green rectangles randomly appearing on the computer screen. The participant's task is to correctly click on the rectangle as quickly as possible.

Tests of visual discrimination

The Penn Line Orientation Test (VSPLOT-24) involves a red line and a blue line both randomly oriented on the computer screen. The participant can click on directional buttons and tries to with rotate the blue line to be exactly parallel to the red line. The participant submits their answer when they think the lines are parallel.

The Penn Emotion Discrimination Test (MEDF-36) involves the participant determining which of two faces in a pair is exhibiting more of the specified emotion. Of the presented questions, a subset involves subtle differences (10% to 20%) between the two faces; the subset score was used to gauge whether the participant can notice slight graphical differences.

Tests of recall

The Penn Facial Memory Test (CPF) involves the participant tasked with memorizing twenty facial photos. The test presents a series of photos, and the participant answers whether the presented photo was in the previously memorized set.

The Short Visual Object Learning Test (SVOLT) has the same structure as the facial memory test above, but with partially shaded geometric shapes instead of faces.

The Short Visual Object Learning Test Delayed (SVOLT-D) asks participants to recall the first memorization round with no review, to see if the participant has passively remembered the shape set after engaging their cognition in multiple different tasks.

Since all three involve the participant responding with certainty or uncertainty, we combined their level of certainty in their responses as a separate measure.

In addition, the Short Letter N-Back 2 (SLNB2) asks participants to respond to a series of flashing letters on the screen with a button press if it meets a certain condition and no response if the stimulus does not satisfy the condition. The first subtest (0-back) is a test of inhibition control, but the subsequent two subtests (1-back and 2-back) rely on the participant memorizing letters that showed up prior to the current one displayed.

Tests of abstract reasoning

The Penn Conditional Exclusion Test (PCET) involves participants guessing which in a series of shapes is the odd one out. The participant receives feedback on each guess, and the implied rule changes over rounds.

The Penn Matrix Reasoning Test (PMAT-24) presents participants with a matrix of shaded shapes with one empty spot and asks the participant to choose from a set of choices which shaded shape best completes the matrix.

The Abstraction, Inhibition, and Memory Test (AIM) involves participants choosing which pair of colored shapes they think the presented colored shape belongs to. The participant receives feedback on each answer, and the implied rule can change over rounds.

Tests of sustained attention

The Penn Continuous Performance Test (PCPT) tasks participants with correctly reacting to valid alphanumeric characters among the flashing characters and character-adjacent digital displays. The participant needs to sustain a Go-No Go state of attentive inhibition for an extended period.

References

1. Gur, R. C. *et al.* Computerized neurocognitive scanning: I. Methodology and validation in healthy people. *Neuropsychopharmacol. Off. Publ. Am. Coll. Neuropsychopharmacol.* **25**, 766–776 (2001).
